# Epidural Anesthesia for Neuromyelitis Optica in Obstetrics: A Case Report and Literature Review

**DOI:** 10.7759/cureus.54966

**Published:** 2024-02-26

**Authors:** Aliki Tympa Grigoriadou, Thalis Asimakopoulos, Christina Orfanou, Aikaterini Melemeni, Athanasia Tsaroucha

**Affiliations:** 1 1st Department of Anesthesiology, Aretaieion University Hospital, National and Kapodistrian University of Athens, Athens, GRC

**Keywords:** devic's disease, labor epidural analgesia, neuromyelitys optica, "epidural anesthesia", devic's neuromyelitis optica

## Abstract

Neuromyelitis optica (NMO), also known as Devic disease, poses unique challenges in obstetrical anesthesia, with limited research available. This case report presents the anesthetic management of a 43-year-old gravida 2 para 1 (G2P1) woman with NMO undergoing labor induction at 39 weeks of gestation. Having received uneventful epidural labor analgesia in her first pregnancy, she faced the delicate decision of neuraxial anesthesia in light of her NMO diagnosis, which was made following her initial pregnancy. Collaborative discussions resulted in the choice of labor epidural analgesia, and an indwelling epidural catheter was placed successfully. An unplanned cesarean section with effective epidural supplementation followed. The case highlights the intricacies of balancing pain relief and neurological risks in NMO patients. Existing literature reflects varying perspectives on neuraxial anesthesia in NMO, with reports both supporting and cautioning against its use. The case aligns with the current view that epidural labor analgesia appears safe in NMO, but conclusive recommendations await larger studies. The decision for neuraxial anesthesia in NMO should be individualized, guided by comprehensive pre-anesthetic counseling and ongoing research developments.

## Introduction

Neuromyelitis optica (NMO), also known as Devic disease, is a rare autoinflammatory central nervous system demyelinating disease associated with serum aquaporin-4 autoantibodies (AQP4-IgG). The spinal cord and optic nerve are primarily affected. Despite sharing many characteristics with multiple sclerosis (MS), NMO is a separate pathophysiological condition with relatively little scientific research on pregnancy and obstetrical anesthesia/analgesia [1,2]. Providing neuraxial (epidural or subarachnoid) anesthesia in patients with NMO is still controversial [3].

The challenges faced in administering anesthesia to these patients require a meticulous understanding to ensure safe and effective care during labor and delivery. The neurologic considerations along with the anesthetic management of a 43-year-old gravida 2 para 1 (G2P1) woman with NMO undergoing cesarean delivery are presented in this article.

This article was previously presented as an e-poster at the Sixth World Congress on Regional Anaesthesia & Pain Medicine (WcRAPM) in Paris, France, on September 8, 2023 [4].

## Case presentation

A 43-year-old, 90 kg, 167 cm, G2P1 woman was admitted for labor induction at 39 weeks of gestation. The patient had a non-reassuring medical history that began during her first pregnancy when she presented with initial neurologic symptoms, including diplopia and facial nerve palsy. These symptoms were initially diagnosed as brain stem syndrome in remission. Notably, during her first pregnancy, the parturient received uneventful epidural labor analgesia for pain management.

**Figure 1:**
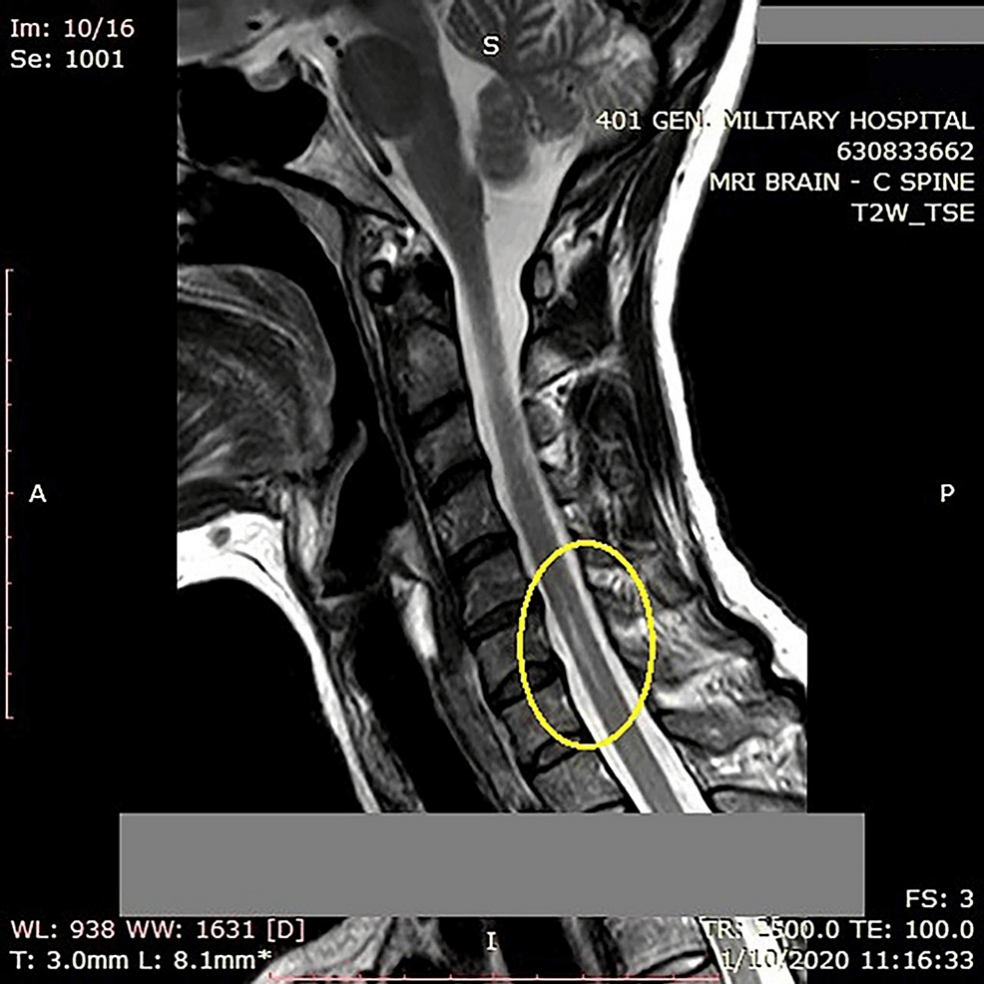
Sagittal T2-weighted magnetic resonance imaging (MRI) of the spine showing a hyperintense region in the spinal cord, consistent with syringohydromyelia, extending from C6 to C7 with a cephalocaudal diameter of 16 mm

Approximately a year later, she was diagnosed with NMO, which was further confirmed by positive testing for AQP4-IgG antibodies. Upon admission at the maternity clinic, the parturient was symptom-free with an unremarkable neurological examination. Magnetic resonance imaging (MRI) revealed a hyperintense region in the spinal cord, consistent with syringohydromyelia, extending from C6 to C7 with a cephalocaudal diameter of 16 mm (Figure 1). Given this diagnosis, the potential risks associated with epidural analgesia in the context of her neurological condition were thoroughly evaluated. The primary concern was to balance the risk of potential neurological symptom exacerbation following the labor epidural with the risk of a disease relapse induced by the stress of labor.

Collaborative discussions between the anesthesiologist and obstetrician were initiated to comprehensively communicate the planned procedure and its associated risks to the patient. After thorough counseling and consideration of the most recent recommendations for this category of patients [1], the parturient opted for labor epidural analgesia.

The patient’s coagulation profile was found to be normal allowing us to proceed safely to regional anesthesia application. With the patient positioned in the left lateral decubitus position, an 18-gauge Tuohy needle was inserted at the L3-L4 intervertebral space; the epidural space was found using the loss-of-resistance technique. A polyamide catheter was easily introduced and placed at the 11-cm catheter mark. A test dose of 2.5 mL lidocaine 2% was injected. After catheter placement, the patient was turned supine with left uterine displacement, and 8 mL of ropivacaine 0.2% was administered, resulting in the establishment of an adequate sensory block for pain relief.

Upon block assessment with the cold sensation test, a unilaterally denser sensory block was noted; it was attributed to enhanced sensitivity to the local anesthetic, presumably deriving from spinal cord damage, as the length of catheter insertion into the epidural space was checked and found to be optimal. It should also be noted that the parturient received only one epidural dose in the delivery suite as her analgesia needs were minimal.

Ultimately, several hours later, the parturient underwent an unplanned cesarean section due to obstetric indications. The epidural anesthesia was effectively topped up for the surgical intervention, ensuring a pain-free and stable perioperative course. Α T4 sensory block was established by ropivacaine 0.75%, 12 mL, and fentanyl 50 mcg. A healthy male infant with Apgar scores of 9 and 10 at the first and the fifth minutes, respectively, was born.

Upon postpartum evaluation, the patient reported no worsening of her pre-existing neurological issues. She was contacted via telephone at 3, 6, and 12 months after discharge, and she reported no change in her symptoms.

## Discussion

Managing anesthesia in patients with NMO demands a delicate balance between providing effective pain relief and minimizing the risk of exacerbating neurological symptoms. Literature indicates varying responses to local anesthetics in patients with demyelinating disorders, and particularly for NMO, there are two reports [3,5] that correlate spinal anesthesia with the development or revealing of the disease. Hosseini et al. raised concerns about a possible cause-effect relationship between neuraxial anesthesia with bupivacaine and NMO [5]. Additionally, Facco et al. suggested the unmasking of latent NMO in a pregnant patient who underwent subarachnoid anesthesia for a cesarean delivery [3]. It was hypothesized that the patient may have experienced severe tetraparesis due to her demyelinated nerves' sensitivity to local anesthetic toxicity. According to this report, spinal anesthesia might only serve to reveal the underlying NMO rather than inducing it [3].

Anesthesiologists tend to avoid regional anesthesia in NMO patients [6,7]. Sadana et al. published their successful anesthetic management of a patient with NMO who underwent cesarean delivery under general anesthesia [6]. Similarly, Dusitkasem et al. performed general anesthesia on an NMO parturient undergoing cesarean section, given the risk of deteriorating neurological symptoms and provoking disease relapse after neuraxial anesthesia [7].

On the other hand, there have also been other cases such as ours, reporting the uneventful use of epidural or subarachnoid anesthesia in parturients with pre-existing NMO [8-10]. Gunaydin et al. performed epidural anesthesia with bupivacaine combined with fentanyl in a 29-year-old paraplegic woman suffering from NMO who was scheduled to undergo a caesarian section and achieved an uncomplicated course of anesthesia [8]. Likewise, Chang et al. successfully performed epidural anesthesia on a patient with NMO who underwent induction of labor for intrauterine fetal demise (IUFD) with ropivacaine and fentanyl, reporting no exacerbation in her pre-existing neurological symptoms during the postpartum assessment [9]. Finally, a case of a primigravida undergoing cesarean delivery at 32 weeks of gestation due to an acute exacerbation of NMO was reported by Greene et al., who opted for subarachnoid anesthesia [10]. No evident anesthetic-related problems were reported, apart from increased intraoperative hyperalgesia which was controlled with inhaled nitrous oxide/oxygen [10].

All these reports are in agreement with the recent recommendations of the French Multiple Sclerosis Society that encourage anesthesiologists to practice anesthesia in NMO patients as in the general population [1].

Undoubtedly, in the present case report, the history of uneventful previous epidural anesthesia significantly exerted influence on our decision. Regarding MS, which shares common characteristics with NMO, as already stated, there is solid data that no clear evidence of a causal relationship between neuraxial anesthesia and disease progression can be firmly established. Case reports, case series, and large studies illustrate that neuraxial anesthesia is considered a safe and widely accepted anesthetic management of the MS population [11].

## Conclusions

In conclusion, most of the available data on obstetrical analgesia in NMO come from small retrospective studies and case reports. Nevertheless, no clear indication of adverse events or relapses following obstetrical neuraxial anesthesia seems to exist. This case report contributes to the existing literature by reinforcing the current view that epidural labor analgesia neither causes nor exacerbates NMO symptoms. Despite the aforementioned points, firm conclusions cannot be drawn due to the lack of larger studies and randomized controlled trials. Finally, the decision to proceed with neuraxial anesthesia in a parturient with NMO should always be made on an individualized basis after thorough pre-anesthetic and obstetric counseling.
